# Blood flow Restriction training After patellar INStability (BRAINS Trial)

**DOI:** 10.1186/s13063-022-06017-1

**Published:** 2022-01-28

**Authors:** Benjamin D. Brightwell, Austin Stone, Xiaojuan Li, Peter Hardy, Katherine Thompson, Brian Noehren, Cale Jacobs

**Affiliations:** 1 University of Kentucky, 740 S Limestone, Suite K401, Lexington, KY 40536-0284 USA; 2 Cleveland Clinic, Cleveland, USA

**Keywords:** Patellar instability, Blood-flow restriction training, Rehabilitation, Randomized controlled trial

## Abstract

**Background:**

Patellar instability is a common and understudied condition that disproportionally affects athletes and military personnel. The rate of post-traumatic osteoarthritis that develops following a patellar dislocation can be up to 50% of individuals 5–15 years after injury. Conservative treatment is the standard of care for patellar instability however, there are no evidence-informed rehabilitation guidelines in the scientific literature. The purpose of this study is to assess the effectiveness of blood-flow restriction training (BFRT) for patellar instability. Our hypotheses are that this strategy will improve patient-reported outcomes and accelerate restoration of symmetric strength and knee biomechanics necessary to safely return to activity.

**Methods/design:**

This is a parallel-group, superiority, randomized, double-blinded, placebo-controlled clinical trial at the University of Kentucky, sports medicine clinic that aims to recruit 78 patients with acute patellar dislocations randomly allocated into two groups: (1) sham BFRT and (2) BFRT. Both groups will receive the current standard of care physical therapy 3 times per week for up to 9 weeks. Physical therapy sessions will consist of typical standard of care treatment followed by BFRT or sham BFRT. Primary outcomes include the Norwich Patellar Instability Scale, quadriceps strength, and imaging and biochemical biomarkers of cartilage degradation.

**Discussion:**

The current standard of care for non-operative treatment of patellar instability is highly variable does not adequately address the mechanisms necessary to restore lower extremity function and protect the long-term health of articular cartilage following injury. This proposed novel intervention strategy uses an easily implementable therapy to evaluate if BFRT significantly improves patient-reported outcomes, function, and joint health over the first year of recovery.

**Trial registration:**

Blood Flow Restriction Training, Aspiration, and Intraarticular Normal Saline (BRAINS) NCT04554212. Registered on 18 September 2020.

## Background

Patellar dislocations are a common and understudied condition that disproportionally affects athletes and military personnel [1]. The rate of having a patellar dislocation is greater than having an anterior cruciate ligament (ACL) tear in this population [1]. Regardless of intervention, 30% to 50% of those who sustain a primary dislocation experience continued pain and the sensation of instability [2]. A significant long-term concern is the rate of post-traumatic osteoarthritis that develops following a patellar dislocation which can be up to 50% of individuals within 5–15 years after injury [3]. Nonoperative treatment is currently the standard of care following most first-time dislocations; however, 31% of patellar instability patients treated conservatively suffer a re-dislocation [4]. A single patellar dislocation increases the lifetime risk of post-traumatic osteoarthritis (PTOA) and recurrent dislocations markedly increase this risk [3, 5]. Therefore, there is an urgent need to minimize the incidents of recurrent patellar dislocations and PTOA sequela which safely allows an earlier return to sport or work while also limiting long-term functional impairments and reduced quality of life.

While nonoperative treatment is most commonly employed for patellar instability, specific evidence-informed rehabilitation guidelines are sparse [4]. One modality that could potentially improve treatment for those with patellar instability is blood flow restriction training (BFRT). BFRT involves the use of an inflatable cuff applied to the thigh to slow arterial blood flow and eliminate venous return as subjects exercise with a relatively low resistance. It is thought that the increased metabolic stress and mechanical tension during BFRT act to stimulate various metabolic pathways leading to muscle hypertrophy [6–8]. There is evidence supporting the premise that BFRT will result in improved quadriceps and hip strength without the need for high-intensity resistance exercise [9–11]. Due to the nature of patellar instability, patients may not tolerate high-intensity quadriceps exercises early in the rehabilitation process thus delaying the recovery of strength. BFRT may allow them to receive the same training benefits as if they were training under high loads. Achieving greater muscle strength earlier after injury may result in improved lower extremity biomechanics, improved functional capacity, and an accelerated recovery.

The purpose of this double-blind randomized clinical trial is to assess whether BFRT in addition to standard physical therapy will improve patient-reported outcomes and accelerate return to work or sport through the earlier restoration of symmetric strength and knee biomechanics, and reduce early cartilage degradation for those with patellar instability.

## Methods/design

### Aims

The overarching rationale is that BFRT will expedite the return to work or sports, minimize muscle impairment, and ultimately slow cartilage degradation over the first year following patellar dislocation. This strategy will improve patient-reported outcomes and accelerate restoration of symmetric strength and knee biomechanics necessary to safely return to activity. We will test the following specific aims:

*AIM 1*: Determine if BFRT improves patient-reported outcomes and lessens the time to return to work or sport following patellar instability.

*Hypothesis:* BFRT will result in significantly improved responses on the patient-reported Norwich Patella Instability, visual analog scale (VAS) for Pain, Cincinnati Occupational Rating Scale, Tegner activity scores, and an earlier return to work and/or sport.

*AIM 2:* Assess whether BFRT results in more symmetric quadriceps strength and knee biomechanics following patellar instability.

*Hypothesis:* BFRT will result in improved quadriceps strength and knee biomechanics (knee moments and excursions).

*AIM 3:* Compare progressive changes in cartilage degradation, muscle architecture, and inflammatory profiles between those treated with or without BFRT over the first 2 years.

*Hypothesis:* BFRT will reduce degradative changes on Magnetic Resonance Imaging (MRI) as well as serum and urinary biomarkers of inflammation and cartilage degradation.

### Participants

Participants will be males and females recruited from the University of Kentucky Sports Medicine Clinic. A sample of 78 patients with patellar instability will be recruited. Potential study participants will be identified by members of the research team during regularly scheduled office visits with 6 physicians at the UK Orthopaedic and Sports Medicine Center. After being identified as a potential study participant, the patient will meet with a research assistant. Provided that the patient meets all inclusion and exclusion criteria, a clinical research coordinator will begin the process to obtain informed consent. Before enrollment, each subject will provide written informed consent. An American Board of Family Medicine-certified physician with a Certificate of Added Qualifications in Sports Medicine, aboard-certified orthopedic surgeon, or a licensed physical therapist will make the diagnosis of patellar instability utilizing the following criteria: patients’ report of at least one episode of patellar instability within the past 3 months, a positive patellar apprehension test during clinical examination, and no evidence on MRI or x-ray of loose body formation or osteochondral defects, avulsion, or osteochondral defect that would require the need for surgical intervention (Table 1).

**Table 1:** Inclusion and exclusion criteria for the BRAINS clinical trial for patients with patellar instability

Inclusion criteria	
Diagnosis of traumatic patellar dislocation (either primary or recurrent) determined by an American Board of Family Medicine-certified physician with a Certificate of Added Qualifications in Sports Medicine, a board-certified orthopedic surgeon, or a licensed physical therapist utilizing clinical examination, radiographic imaging, and patients’ reports of instability	
Age 14 to 40 years	
Skeletally mature with closed growth plates visualized by radiograph	
A score of 80 or more on the Sports Activity Scale, which corresponds to participating in “running, twisting, turning (tennis, racquetball, handball, ice hockey, field hockey, skiing, wrestling)” at least 1–3 times per week	
Desire to resume pre-injury activity level	
Exclusion criteria	
Concomitant osteochondral lesion requiring surgical fixation	
Radiographic evidence of osteoarthritis (≥ Kellgren-Lawrence Grade 2)	
Previous ipsilateral or contralateral knee surgery	
Most recent instability event more than 3 months before enrollment	
History of intra-articular injection into either knee within 3 months	
History of any inflammatory disorder	
Body mass index > 35 kg/m^2^	
Diabetes or uncontrolled hypertension	
Varicose veins or a history of personal or immediate family history (parental or sibling) of deep vein thrombosis	

### Design

This is a parallel-group, superiority, randomized, double-blinded, placebo-controlled clinical trial that begins in the winter of 2022 and will continue for 4 years. This study will take place at the University of Kentucky, and has been approved by the University of Kentucky IRB (IRB #56541, ClinicalTrials.gov Identifier: NCT04554212). Any changes to the protocol will be first reviewed by the local IRB and then communicated to the entire study team upon approval.

### Randomization, group allocation, and blinding

Randomization: Following informed consent and after successfully being screened into the study based on exam findings, subjects will be randomized to one of two groups. The randomization schedule will be made a predesignated unblinded study team member that will not be involved in data collection or analysis. Using a random number generator, allocation of to either the BFRT or sham BFRT group will be by permutated random block size, ensuring that approximately equal numbers of subjects will be treated with drug or placebo in the unlikely event that the study will need to be terminated prematurely. Should a subject discontinue the study for any reason prior to the final follow-up visit, then the collected data will be evaluated but not included in the analysis and a replacement subject will be enrolled in the study. As such, the randomization schedule will be prepared for 100 potential participants despite having a target enrollment of 78 participants to ensure adequate power.

Blinding: The team member that prepared the randomization schedule will meet with the pre-specified unblinded study personnel prior to the site initiation visit. The unblinded personnel will then prepare sealed envelopes that contain each subject’s group assignment. All assessments and data analyses will be performed by a separate individual to the person applying the BFRT unit to ensure blinding. The study statistician will generate a randomization plan using SAS or R to assign subjects to groups. Randomization will occur by sex and be in sets of 2 subjects to ensure adequate distribution of all groups across the collection period. Patients will be randomized into one of the two following treatment groups:

1. Standard of care physical therapy + sham BFRT
2. Standard of care physical therapy + BFRT

Should an investigator become inadvertently unblinded, the medical officer and principal investigator will determine the extent of the unblinding and potential ramifications. If necessary, the investigator’s role may need to be altered to only include tasks affiliated with unblinded personnel. Should a patient become inadvertently unblinded, that patient’s data will continue to be collected for safety purposes; however, the data will not be used for the final analyses and a replacement subject will be enrolled. Unblinding of a subject(s) group assignment will be documented and will include an explanation of why the study intervention was unblinded. Following data lock, group assignment can be unblinded and the unblinded information can be sent to the study participants at the discretion of the principal investigator.

### Interventions

All groups will receive current standard of care physical therapy from a licensed physical therapist. Physical therapy sessions will consist of typical standard of care treatment followed by BFRT or sham BFRT. The standard of care portion of the session will consist of brief subjective and objective screening followed by a structured treatment paradigm consisting of pain modulation, neuromuscular re-education, dynamic warm-up, range of motion and mobility, progressive strengthening, and power training with an emphasis on the lower extremities and trunk musculature. The standard physical therapy portion of the treatment session will last approximately 40 min. Interventions for pain modulation, range of motion, and neuromuscular re-education, balance, and gait training will be utilized as needed to address individual subjects’ specific needs at the time of screening based on the severity of the injury and with the goal of adequately preparing them to return to their sport of choice.

### Blood flow restriction training

**Fig. 1:**
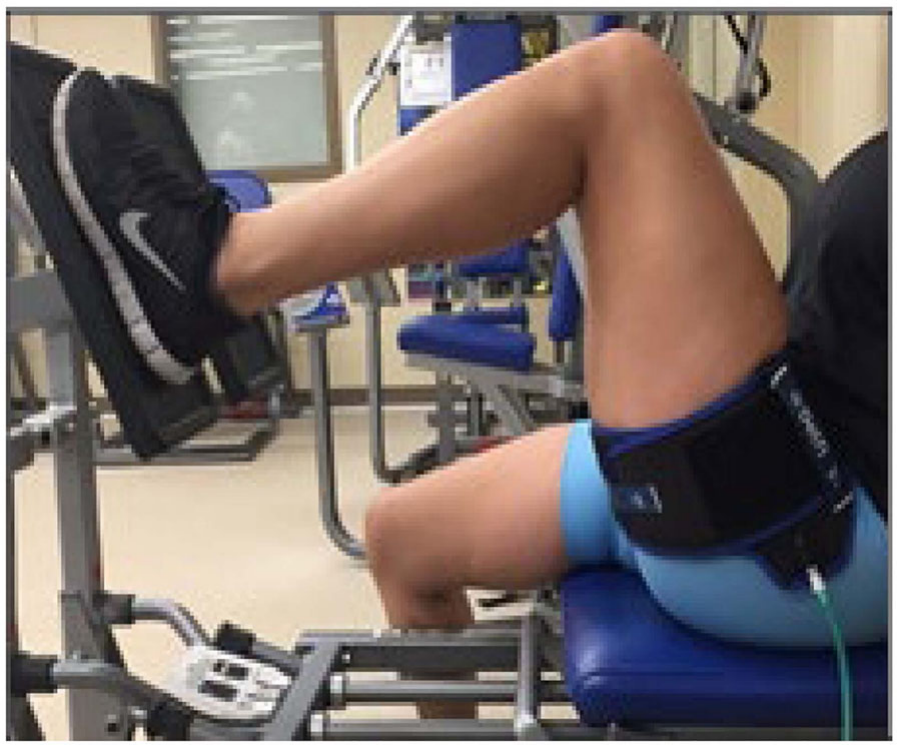
Blood flow restriction training cuff placement. The BFRT band is applied to the upper thigh of the subject’s leg. Pressure is continuously regulated by the controller unit during training

Two brands of blood flow restriction units will be used (brand names not included in this text to avoid the potential for subjects to become unblinded). Subjects will be split into groups as previously described where brand 1 will be used as sham BFRT and the intervention group will use brand 2. All quadriceps-focused exercises will be performed with the bands inflated. As directed by the manufacturer’s instructions, the bands will be placed on the proximal thigh as close to the femoroacetabular joint as possible (Fig. 1). Limb arterial occlusion pressure will be constantly monitored and adjusted by the unit to maintain the desired occlusion pressure throughout the duration of exercise. Consistent with our current trial assessing the efficacy of BFRT for ACL reconstruction patients (NCT03364647), participants in all groups will perform the same exercises with an emphasis on quadriceps and hip strengthening, and for those randomized to the active BFRT groups, each BFRT session will last approximately 20 min [12]. All subjects will attend 3 visits per week for 8 weeks divided in to 4 phases.

The active BFRT group will have the arterial blood flow occlusion pressure set per manufacturer instructions to achieve the desired limb occlusion pressure (LOP), whereas the sham BFRT group will have restriction pressure set at a sub-clinical level of occlusion. Occlusion personalized tourniquet pressure (PTP) will be reassessed each visit by rechecking the LOP. Each subject will perform a block of 4–5 quadriceps and hip-focused exercises each lasting approximately 5 min for a total of 20 min (exercises: seated single leg press, seated single leg knee extension, single leg forward step-ups progressed to forward step downs when tolerated, and resisted double leg squats). Exercise intensity will be adjusted between groups to accommodate the achievement of volitional fatigue and an acceptable sensation of discomfort during exercise. Cuffs will be deflated for a minimum of 1 min between exercises. The quadriceps and hip strengthening exercises with the cuff donned will be performed at the end of the rehabilitation session with the intention of reducing the impact of fatiguing the muscles for the standard physical therapy portion of the treatment session.

For all subjects, rating of perceived discomfort and rating of perceived effort will be recorded from separate 11-point Likert-type scale (0 no discomfort or effort to 10 maximal discomfort or effort) for each BFRT exercise. Emphasis will be placed on distinguishing between effort (the amount of physical energy being given to the exercise) and discomfort (the physiological and unpleasant sensations associated with the exercise), with the goal of maximizing effort. All exercises should be challenging and/or hard to perform; however, subjects need to maintain proper form, as determined by the treating physical therapist, to ensure appropriate quadriceps engagement.

Criteria for progression of resistance or exercises include (1) no excessively aberrant movement causing pain due to fatigue of desired muscle group(s), (2) no excessive form deviation where the desired muscle group is no longer the primary mover completing the desired task, (3) no concordant pain > 4/10, (4) ability to complete delineated sets and repetitions without pausing within a set, and (5) able to complete 2 or more repetitions than delineated during the last set of the exercise.

Criteria for clearance to return to sport include (1) negative moving patellar apprehension test, (2) a score of 0 indicating no effusion with the stroke test, (3) at least 0 degrees of knee extension active range of motion of the involved knee, (4) knee flexion active range of motion within 5 degrees of the uninvolved knee, (5) < 4/10 numeric pain rating with activity, and (6) 90% limb symmetry index as measured with isometric knee extension peak torque and the 60-s step down test.

### Treatment adherence

Treatment adherence is the extent to which a therapist uses interventions prescribed by a protocol. To maximize adherence and treatment fidelity, electronic logs of all the exercises per session will be kept on a custom Microsoft Access database. Adherence to the treatment protocol will be audited by an external physical therapist regularly throughout the duration of the study. We will monitor session attendance using a diary log with participant ID numbers and session dates to track the percentage of attendance and to account for absenteeism and reasons for missed sessions. We will make every effort to deliver the full treatment, but we expect some variation in treatment delivery dose as well as treatment drop-out. Reasons for attrition will be assessed with an open-ended question for enrolled participants who withdraw.

### Outcome measures

Primary outcomes include the Norwich Patellar Instability Scale [13] collected at the time of enrollment, 4 weeks, 9 weeks, 6 months, and 12 months after enrollment, isometric and isokinetic quadriceps strength at 1 week, 4 weeks, 9 weeks, 6 months, and 12 months after enrollment, MRI T1ρ and T2 relaxation times 1 week, and 12 months enrollment, and biochemical biomarkers which will include urinary C-terminal cross-linked telopeptide type II collagen (CTXII) and a neoepitope of type I and type II collagen cleavage (C1,2C) at the time of enrollment, 4 weeks, 9 weeks, 6 months, and 12 months after enrollment. Secondary outcomes include the VAS for pain [14], Cincinnati Occupational Rating Scale [15], Tegner Activity Scale [16], and the time to returning to work and/or sporting activities, hip muscle strength (hip abduction, hip external rotation), and knee biomechanics (knee extensor moment, knee flexion excursion, knee adduction moment, knee adduction excursion) during walking, running, and anterior step-downs 1 week, 9 weeks, 6 months, and 12 months after enrollment. Data will be captured using REDCap and will be reviewed on a quarterly basis by an unblinded member of the study team to ensure data quality and completeness (Table 2).

**Table 2:**
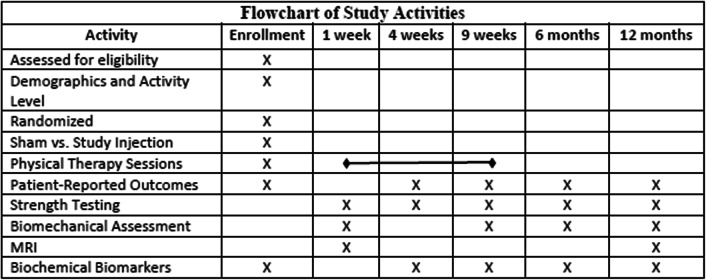
Flowchart of study activities

### Statistical methods

The primary outcome variable for Aim 1 is the Norwich Patellar Instability Scale. Utilizing the standard deviation of 18.6 points from our pilot results, a sample size of 34 subjects per group will be adequately powered (80%) to detect a moderate effect size between groups difference (13 points difference, effect size = 0.7). To protect against up to 15% of subjects being lost to follow-up, an additional 5 subjects will be enrolled per group (39 subjects per group; 78 total). Based on our previous pilot work, a sample size of 34 patients per group will also be more than 80% powered to detect group differences in the primary outcome variables for both Aim 2 (quadriceps strength and knee biomechanics) and Aim 3 (T1ρ relaxation time).

Since all endpoints are quantitative and longitudinal data is collected, repeated measures analysis of covariance (ANCOVAs) will be used to assess differences in scales across the two treatment groups (BFRT vs. sham BFRT). The repeated measures ANCOVAs will adjust for demographic covariates (e.g., age, sex, and race) as well as look for differences across any other potential confounders (such as first-time vs. repeated instability events). Assumptions such as normality and constant variance will be assessed, and remedial measures will be employed as needed. In addition, interaction effects and/or variable transformations will be explored appropriately. In the case that endpoints are related, multivariate analysis of covariance (MANCOVAs) may be used to consider those that naturally group themselves.

All analyses will be performed with a significance level of 0.05, and appropriate multiple comparisons adjustments will be performed as needed. Categorical variables will be described with counts and percentages and analyzed using appropriate statistical methods, such as hypothesis tests or logistic regression models.

For both primary and secondary analyses, in the case of participants that violate group allocation or do not attend a minimum of 80% of the prescribed physical therapy sessions, we will perform intention-to-treat and per protocol analyses. We will not employ imputation methods in the case of missing data. No interim or other subgroup analyses will be performed.

Participant information will be combined with information from other people taking part in the study. When we write about the study to share it with other researchers, we will write about the combined information we have gathered. Participants will not be personally identified in these written materials. We may publish the results of this study; however, we will keep participant names and other identifying information private. The study information collected from participation in the study may be entered into a secure computer system. The study physician is committed to maintaining the privacy of every study participant and any personal information submitted and follows the principles of the Health Insurance Portability and Accountability Act (HIPAA) of 1996. All data are stored at a secure site behind a firewall, which monitors and protects against unauthorized access. Participant charts and any other items containing confidential items will be stored in a safe place overnight and not left on the desk. Charts will not be left in an area where others might have access to them. Participants should know, however, that there are some circumstances in which we may have to show their information to other people. For example, the law may require us to show their information to a court or to tell authorities if they report information about a child being abused or if they pose a danger to themselves or someone else. Officials of the University of Kentucky, the Department of Defense, members of an independent Data Safety Monitor Board, the Center for Clinical and Translational Science, and Bluegrass Research Consultants may look at or copy pertinent portions of records that identify participants. Participants withdraw from the study or are withdrawn; data collected to that point will be kept.

### Monitoring

We will utilize the standing independent Data Safety Monitoring Board (DSMB) as chartered by the Center for Clinical and Translational Science (CCTS). The DSMB will meet three times per year or as needed and will review participant recruitment, AE’s, side effects, laboratory results, dropouts, protocol violations, and inclusion/exclusion criteria. More frequent meetings will take place if side effects or other problems are prevalent. Should an adverse or serious adverse event occur, the research participant will be followed by physicians, registered nurses, and other research staff members for the duration of the participant’s hospitalization. Routine care will be provided by hospital staff. Emergency medical equipment, medications, and supplies will be at the physician’s disposal should the participants have an acute untoward reaction.

## Discussion

Currently, scientific evidence regarding the medical management and treatment of patellar instability is sparse. As such, the standard of care for non-operative treatment is highly variable does not adequately address the mechanisms necessary to restore lower extremity function and protect the long-term health of articular cartilage and surrounding musculature following injury. A significant need remains to identify novel therapies that successfully restore function and preserve joint integrity. The goal of this trial is to evaluate if augmenting standard physical therapy with BFRT significantly improves patient-reported outcomes, function, and joint health over the first 2 years of recovery.

Blood-flow restriction training is a promising modality for patients who suffer from patellar instability. We have previously shown that these individuals have clinically important impairments in quadriceps and hip strength as well as altered knee biomechanics compared to healthy volunteers [17]. Increased quadriceps strength has been shown to be protective against patellofemoral cartilage loss as well as improved pain and function [18]. Hip weakness and increased knee abduction angles may place the patient at greater risk for repeated lateral patellar dislocation or subluxation and increase the compressive and shear forces born by the articular cartilage [18, 19]. BFRT allows the subject to exercise with light resistance to derive similar training benefits seen with higher-intensity resistance exercises (Fig. 1) [6–10, 20–23]. Potentially, with BFRT the negative morphological adaptations of hip and thigh musculature that result in abnormal lower extremity biomechanics can be addressed, while compressive and shear forces born by the patellofemoral articular cartilage are minimized thereby promoting recovery [11, 19].

We seek to address the shortcomings of the current treatment strategies by specifically addressing progressive cartilage degradation, muscle weakness, and functional impairment for those with patellar instability. Current physical therapy protocols do not adequately restore quadriceps or hip muscle strength resulting in altered knee biomechanics. The combination of a pro-inflammatory intra-articular environment and persistent biomechanical deficits undoubtedly contribute to the high rate of PTOA for those with patellar instability. By potentially addressing both biologic and biomechanical mechanisms of PTOA, the proposed interventions may alter the progression of PTOA in this high-risk patient population. The long-term impact of this study is that the progression of PTOA may be delayed or potentially even avoided. These results may then impact the treatment paradigms for other acute conditions such as anterior cruciate ligament injuries, shoulder dislocation, and/or ankle sprains.

## Trial status

This is protocol version 2.0 (December 15, 2021). Patient recruitment will begin in January 2022 with recruitment ending December 2023.

## Data Availability

Data will be collected in a protected database with an unidentified ID number provided for each participant. All authors of the current manuscript will have access to the final dataset. Participant-level data are available on request by sending an email to Cale Jacobs. All data will be available for 5 years after the relevant publication.

## Abbreviations

BRAINS: Blood flow Restriction training, Aspiration, and Intra-articular Normal Saline
BFRT: Blood-flow restriction training
ACL: Anterior cruciate ligament
PTOA: Post-traumatic osteoarthritis
IL-1a: Interleukin-1a
MMP-3: Matrix metalloproteinase-3
VAS: Visual analog scale
MRI: Magnetic resonance imaging
LOP: Limb occlusion pressure
PTP: Personalized tourniquet pressure
ANCOVAs: Analysis of covariance
MANCOVAs: Multivariate analysis of covariance
